# Signal Response Sensitivity in the Yeast Mitogen-Activated Protein Kinase Cascade

**DOI:** 10.1371/journal.pone.0011568

**Published:** 2010-07-23

**Authors:** Craig J. Thalhauser, Natalia L. Komarova

**Affiliations:** 1 Department of Mathematics, University of California Irvine, Irvine, California, United States of America; 2 Department of Mathematics, University of California Irvine, Irvine, California, United States of America; Fondazione Telethon, Italy

## Abstract

The yeast pheromone response pathway is a canonical three-step mitogen activated protein kinase (MAPK) cascade which requires a scaffold protein for proper signal transduction. Recent experimental studies into the role the scaffold plays in modulating the character of the transduced signal, show that the presence of the scaffold increases the biphasic nature of the signal response. This runs contrary to prior theoretical investigations into how scaffolds function. We describe a mathematical model of the yeast MAPK cascade specifically designed to capture the experimental conditions and results of these empirical studies. We demonstrate how the system can exhibit either graded or ultrasensitive (biphasic) response dynamics based on the binding kinetics of enzymes to the scaffold. At the basis of our theory is an analytical result that weak interactions make the response biphasic while tight interactions lead to a graded response. We then show via an analysis of the kinetic binding rate constants how the results of experimental manipulations, modeled as changes to certain of these binding constants, lead to predictions of pathway output consistent with experimental observations. We demonstrate how the results of these experimental manipulations are consistent within the framework of our theoretical treatment of this scaffold-dependent MAPK cascades, and how future efforts in this style of systems biology can be used to interpret the results of other signal transduction observations.

## Introduction

The yeast pheromone response system is one of the first signal transduction systems to be identified and studied in detail [1]–[3]. The system responds to a mating factor secreted by a nearby cell of opposite type. The factor binds to and activates a G-protein coupled receptor, which in turn activates a heterotrimeric G protein, which is responsible for activating the kinase cascade. This cascade is homologous to many mammalian systems of the mitogen activated protein kinase (MAPK) family. These pathways generally consist of two or three steps, where each step involves the activation of a protein kinase, which in turn activates the next enzyme in the system. Typically, each enzyme requires two distinct phosphorylation events in order to become fully active.

In the yeast system, G protein activation leads to the activation of a MAPKKK, Ste11. Ste11 activates the MAPKK Ste7, which has two possible target MAPKs, Fus3 and Kss1 [1]. Both of these MAPKs are induced upon pheromone stimulation. Kss1, but not Fus3, can also be activated via stress and invasive growth signals. The specificity for Fus3 activation by pheromone alone is thought to be provided by a scaffolding protein, Ste5, which binds Fus3, Ste7 and Ste11 along with other elements of the pheromone response pathway [1], [4], [5]. While Ste5 has no catalytic activity of its own, its function is nonetheless necessary for successful response to the pheromone signal.

Scaffolds such as Ste5 have been a subject of extensive theoretical and empirical investigations, much of the work focusing on how the scaffold controls the output response of its pathway [6]–[9]. These responses are generally classified as either ultrasensitive or graded [10]. An ultrasensitive response is one in which little downstream signal response–in this case, Fus3 activation–is observed until the activating signal reaches a threshold. At levels of activation near and above the threshold, the level of response quickly rises to its maximum possible level. This ultrasensitive response (also called a biphasic response) stands in contrast to a graded response, in which increases in activation signal over a wide range of concentrations lead to a concomitant increase in signal response. The type of output response governs whether the signal engages an all-or-nothing response in the cell for critical changes in cell fate such as mating (yeast) or the activation of mutually exclusive genetic programs such as proliferation or differentiation (higher eukaryotes) [11]. Thus understanding how a cell generates a biphasic signal response becomes important to the understanding of the regulation of these cell fate decisions.

Recently, several studies have shown that the yeast Ste5 scaffold plays an important role in modulating the ultrasensitivity of the Fus3 response to pheromone. These reports have shown that the scaffold-dependent Fus3 response is ultrasensitive, whereas the scaffold-independent response of Kss1 is graded [12]. These empirical results were quite startling, as they are in contradiction with several past theoretical investigations into MAPK cascades–both with and without scaffolds [8], [13]. For example, the model of Huang and Ferrell [13], based upon the double phosphorylation activation system common to MAPK cascades and involving no scaffold, demonstrated that for parameter regimes which include mammalian cascades, the system shows a strong and robust biphasic nature, especially in the final kinase of the system. Levchenko *et al* [8] modeled the MAPK cascade in the presence of a scaffold (again based upon a mammalian system, the MP-1 scaffold), under the assumption that a scaffold-bound enzyme could perform multiple catalytic reactions in a processive (bind-catalyze-catalyze) rather than distributed (bind-catalyze-release-rebind-catalyze) mechanism. These systems showed a graded response to signal strength. The system could be modified to create a biphasic output; however, this output came at the cost of many orders of magnitude loss of maximum output signal strength, meaning these modifications were practically impossible.

To summarize, past theoretical investigations into scaffold-free MAPK systems demonstrate a tendency towards a biphasic response, while systems involving a scaffold show in theory a strong and robust graded response, which is contrary to the recent experimental findings. To address this discrepancy between theory and experimental results, we devise a new model of the yeast pheromone response system. This model is tailored to several experimental results from the recent literature, and we demonstrate how the apparent conflict can be resolved by examining how the kinetic binding parameters influence the signal response output.

This work is organized as follows. First, we revisit the classic MAPK cascade in the absence of scaffold and make a detailed investigation of its output signal characteristics. Then, we devise a simplified model of the yeast scaffold-MAPK system and study its dynamics and output response as functions of the kinetic binding parameters of MAPK enzymes to the scaffold. We show how these results are consistent with both prior theoretical studies and current experimental evidence. Finally, we offer several hypotheses, based on our results, which explain how experimental perturbations to this pathway reported in the literature lead to non-obvious changes to the biphasic nature of the transduced signal.

## Methods

### The basic model of MAPK cascade

**Figure 1:**
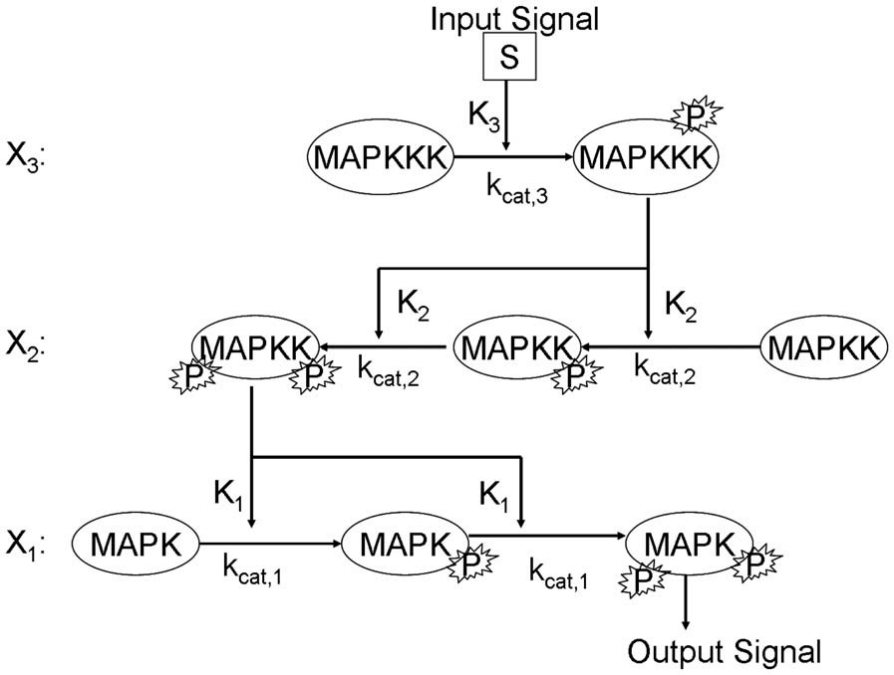
Cartoon representation of the canonical MAPK signaling pathway.

We begin by seeking a better understanding of how the MAPK system transmits its signals in response to a stimulus. We thus start with a simple implementation of the classic MAPKKK
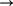
MAPKK
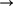
MAPK pathway, found in organisms ranging from yeast to mammals [1], [11], [13]. Each of the arrows represents a double activation step which is assumed to be distributed (i.e. the enzyme must release its substrate and rebind for the second activation step). Such a pathway is shown schematically in figure 1.

The system consists of three enzymes, each of which has one fully activated state and one or more inactive states (table 1). We assume Michaelis-Menton (MM) kinetics; as all of our analyses and simulations will be done to steady state, the assumptions of the MM kinetics are automatically satisfied. We further assume that the phosphorylation events needed to activate MAPKK and MAPK (each requires two) are distributed; that is, the activating enzyme must bind its target, phosphorylate it, release it, then rebind at the second phosphorylation site. The pathway is described by the following equations:


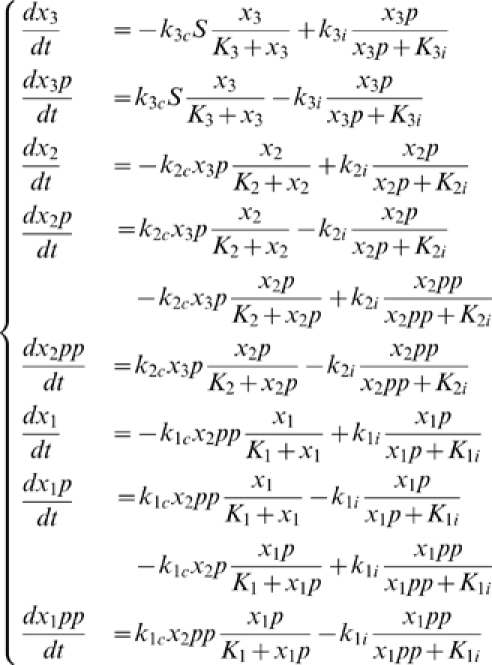
(1)

The parameters for this system may be found in table 2. This model is similar to the canonical system first modeled in [13], with several simplifications which we will use in an analysis of the system response to input signal. The adoption of Michaelis-Menton kinetics for both phosphorylation and dephosphorylation reactions explicitly allows us to remove all enzyme-substrate complexes from consideration, leading to a much more compact system. We can also use the MM binding constant as a reference point to explore different extremes of parameter space, which will allow us to provide bounds for the degree of ultrasensitivity this model is capable of generating.

**Table 1:** State Variables of the MAPK cascade.

Variable	Interpretation
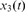	Inactive MAPKKK
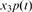	Active MAPKKK
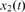	Unphosphorylated MAPKK
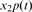	Singly phosphorylated MAPKK
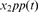	Doubly phosphorylated MAPKK; fully active
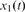	Unphosphorylated MAPK
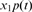	Singly phosphorylated MAPK
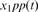	Doubly phosphorylated MAPK; fully active

**Table 2:** Parameter listing for model (1).

Parameter	Meaning	Test value or range	Reference (if available)
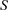	Input signal strength	0–1	[12], [13]
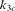	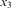 catalytic activation rate by S	0.9–1.5	[21], [25]
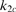	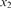 catalytic activation rate by 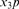	0.75–2.9	[21], [25]
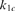	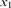 catalytic activation rate by 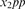	1–5	[21], [25]
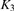	Binding affinity of 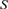 for 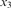	1	[21], [25]
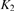	Binding affinity of 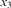 for 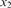	0.01–100	[1], [21], [25]
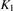	Binding affinity of 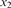 for 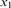	0.01–100	[1], [21], [25]
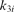	Catalytic inactivation rate of 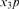	1–2	[12], [21], [25]
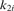	Catalytic inactivation rate of 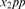	1–2	[12], [21], [25]
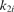	Catalytic inactivation rate of 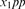	1–2	[12], [21], [25]
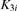	Binding affinity of inactivation for 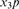	0.15–15	[12], [21], [25]
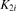	Binding affinity of inactivation for 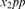	0.15–15	[12], [21], [25]
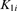	Binding affinity of inactivation for 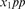	0.15–15	[12], [21], [25]

Binding constants are scaled to total cellular substrate concentration and are thus unitless. Units for the rate constants are sec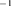.

### Simple Model of the Yeast Scaffold Pathway

We find that model (1) cannot be easily adapted to include the presence of the scaffold, as the number of intermediate steps and complexes in fully active scaffold formation process is quite sizable, even though many of those steps are never detected experimentally. A full system would include a combinatorial set of partially assembled scaffold complexes, and contain both fast-paced and slow reactions. Such complexity makes the description unnecessarily opaque. Instead, we will focus on a relatively simpler system in which we assume equilibrium levels of scaffold complexes have formed, and that the no-signal resting state for the system involves all three enzymes bound to the scaffold, although not in an orientation which is immediately permissive of processive phosphorylation [14].

**Figure 2:**
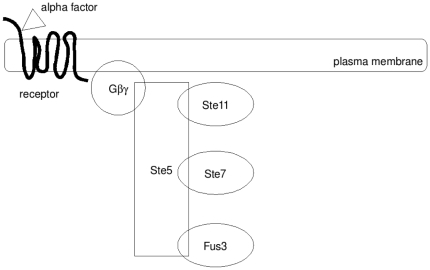
Cartoon representation of the yeast scaffold-MAPK signaling complex.

A signalling event by pheromone through its receptor activates two separate processes, both of which are necessary for proper signal transduction (see figure 2) [1]. Firstly, the Ste5 scaffold binds to a plasma membrane protein complex, the G 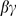 subunit of the G-protein coupled receptor. This binding event can only happen upon G-protein activation. Secondly, the signalled receptor activates a kinase, which can in turn phosphorylate and activate Ste11 MAPKKK. Together, these two processes initiate the full MAPK signal cascade starting with Ste11, to Ste7, and culminating with the effector MAPKs Fus3 and Kss1. It is important to note that only Fus3 is known to bind to the scaffold, while Kss1 remains predominantly cytoplasmic.

In a scaffold-free MAPK cascade described by system (1), the rate-limiting steps are the phosphorylation events. In the presence of a scaffold we hypothesize that the rate-limiting steps are the association events of proteins with the scaffold in an orientation which allows for processive phosphorylation, in accordance with other treatments of scaffold systems. Once the elements of the MAPK cascade are attached to the scaffold, the downstream activation can happen on a faster time-scale. The pertinent reactions are (i) signal-dependent scaffold complex binding to its partner in the plasma membrane, leading to a conformational change in the scaffold bringing the enzymes into alignment with one another; and, (ii) signal-dependent enzymatic activation of the first kinase on the scaffold. The forward and reverse rates for the first reaction are 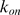 and 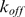, and the catalytic and binding parameters for the second are 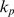 and 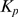 respectively. It is critical to note that the formation of fully competent scaffold can only happen at the plasma membrane, in accordance with the fact that Ste5 must associate with membrane-bound G 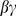 before signal transduction can occur, and that this association involves conformational shifts in the scaffold itself [14], [15]. The activated complex is inactivated by ubiquitous phosphatase activity at catalytic rate 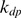 and binding constant 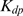. We assume that a fully assembled complex is activated in a single processive step.

Inactivation of the scaffold is a two-step processes. We consider the kinase(s) upstream of the conformational shift are inactivated as a group, and the kinase(s) downstream of the shift are as well. A scaffold with inactive Ste11 but active Fus3 is still considered an active signaling complex. Dephosphorylation of Fus3 on a scaffold which is both properly aligned and has active Ste11 is disregarded, as we assume the Fus3 will immediately be rephosphorylated. However, once a Fus3-active scaffold detaches from its membrane target (and therefore loses its proper alignment) it will remain an active signaler until Fus3 is dephosphorylated, independent of whether or not the upstream enzymes are active or not.

**Figure 3:**
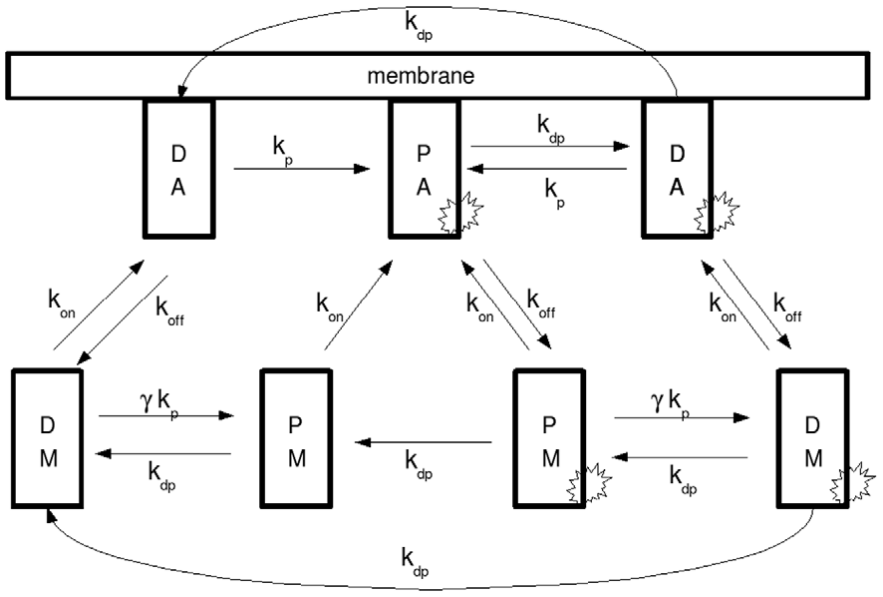
Schematic of activation and deactivation processes in the yeast scaffold-MAPK signaling complex. Names are as given in table 3, with the star representing phosphorylated (and thus active) Fus3. Horizontal arrows represent phosphorylation/dephosphorylation events (P to D and vice versa and inactive Fus3 to active Fus3 and vice versa), while transitions from the inner layer to the membrane layer are binding reactions (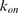 and 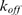).

Our model is graphically presented in figure 3. In this model, there are two necessary independent steps (initial phosphorylation and scaffold alignment) for scaffold activation, leading to four general classes of scaffold complexes: unphosphorylated, unaligned scaffolds; unphosphorylated, membrane-associated aligned scaffolds; phosphorylated, unaligned scaffolds; and phosphorylated, membrane-associated aligned scaffolds. This last class is the only scaffold which becomes fully active for signaling, a process we assume occurs processively and very quickly. Furthermore, scaffolds can exist either with or without active Fus3; those with active Fus3 are considered active signalers. Thus, we have a total of 8 possible classes (combinations of three independent binary markers), of which we model 7 classes. The eighth class, the phosphorylated, membrane-associated scaffold without active Fus3 is assumed to exist for only extremely brief periods of time before its Fus3 is activated. We name these classes based upon the state of each of their markers: (P)hosphorylated or (D)ephosphorylated Ste11; (A)ligned or (M)isaligned Ste7; and active (starred) or inactive Fus3. Thus a scaffold with unphosphorylated Ste11 bound to the membrane with active Fus3 is called 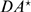. In this diagram, horizontal arrows represent phosphorylation/dephosphorylation events (P to D and vice versa and inactive Fus3 to active Fus3 and vice versa), while transitions from the inner layer to the membrane layer are binding reactions (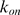 and 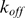).

Finally, we consider two control parameters which guide the flow of signal through the model. The first parameter, 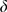, relaxes the restriction that the scaffold must first bind to the plasma membrane in a signal-dependent manner to allow for full activation of the complex. This parameter will allow a comparison between the yeast and mammalian systems, the latter of which do not share the former's binding requirements. The second parameter, 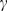, controls to what extent cytosolic scaffolds can be phosphorylated at their Ste11 to initiate activation. A value of 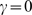 would imply that a scaffold must be bound to its membrane target to be phosphorylated at Ste11, while a value of 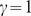 means that cytosolic scaffolds are phosphorylated at the same rate as membrane scaffolds.

With the state variables given in table 3, we have the following system of ODEs describing the various stages of scaffold-MAPK complex assembly and activation:
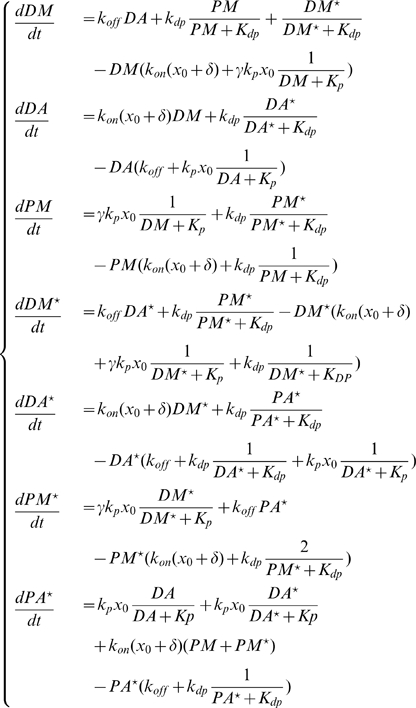
(2)


Parameters values are listed in table 4. This model is significantly less complex than many previous models describing the yeast pheromone/MAPK system, yet we will show it contains all the necessary components to describe the recent experimental investigations into the mechanisms of output signal character.

**Table 3:** State variables for model 2.

Variable	Interpretation
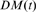	inactive scaffold in cytoplasm with inactive Ste11
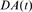	inactive scaffold at membrane with inactive Ste11
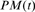	inactive scaffold in cytoplasm with active Ste11
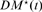	active scaffold in cytoplasm with inactive Ste11
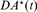	active scaffold at membrane with inactive Ste11
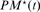	active scaffold in cytoplasm with active Ste11
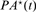	active scaffold at membrane with active Ste11

All variables are scaled to be unitless.

**Table 4:** Parameter listing for model (2).

Parameter	Meaning	Value/Range	Reference (if available)
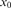	input signal strength	0–1 [12]	
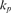	phosphorylation rate	0.03	[12]
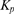	phosphorylation binding constant	0.15	[12]
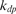	dephosphorylation rate	0.003	[12]
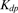	dephosphorylation binding constant	0.15	[12]
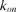	scaffold-signal binding rate	0.01–100	
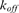	scaffold-signal dissociation rate	1	
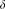	selective activation control switch	0,1	
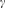	cytosolic scaffold activation control switch	0–1	

Rate constants have units of sec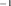, and binding constants have been scaled to be unitless.

While our model takes as its conceptual basis the hypothesis of selective activation of the scaffold, [4], [16], [17] which states MAPKK signal flux to a specific downstream MAPK occurs only upon a secondary signaling event, it is readily generalizable to many different interpretations of the selective activation hypothesis. The model requires as its basic hypotheses merely two separate signaling events, which in this case are one enzymatic and one mass action binding. Both events are necessary before signal transduction becomes permissive. Thus other architectures of the yeast MAPK cascade can be interpreted using this same framework, with new hypotheses added, as demonstrated later in this paper.

## Results

### Hill coefficient analysis of scaffold-free and scaffold-dependent models

#### Analytic considerations of Hill coefficient distributions

We will use model (1) to study signal response sensitivity in the MAPK signaling pathway. The sensitivity is typically measured by fitting the output response of a signaling pathway as a function of the input signal strength (measured either from experiments or numerical simulations) to a standard Hill function:
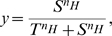
where 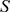 is the signal, 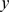 is the response, 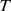 is the threshold point for activation, and 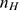 is the Hill coefficient. A higher Hill coefficient yields a stronger biphasic response. Highly ultrasensitive systems, such as the human hemoglobin molecule, have Hill coefficients greater than 2. The reported Hill coefficients for the two output systems of the yeast MAPK pathway are 2.3 (Fus3) and 1.5 (Kss1) [12].

We can analyze the behavior of model (1) by making several simplifying assumptions on the Michaelis-Menton (MM) activation terms. This simplification will allow us to calculate the steady state concentrations of the output product, 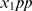, and determine under what conditions it has a biphasic dependence on the input signal strength.

The classic MM equation describes how the rate of formation of product 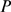 is impacted by the concentration of the substrate 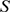 and the enzyme 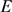 catalyzing the reaction [18]:
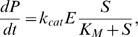
where 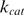 is the catalytic rate constant and 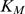 is the binding affinity of the enzyme for the substrate. There are two regimes for which the general MM equation reduces to a simpler form.

Firstly, when 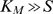, the equation simplifies to a mass-action like term:
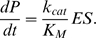
The assumption 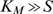 corresponds to the limiting case of very weak enzyme-substrate interactions. Using this simplification, it is possible to calculate the steady state levels of fully active 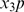, 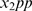 and 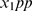 as functions of their activating input signals (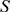, 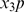 and 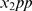 respectively). We apply the weak enzyme-substrate assumption to both phosphorylation and dephosphorylation reactions, and find that the levels of 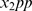 vary as a function:
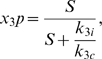
(3)

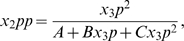
(4)and the levels of 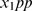 vary as:
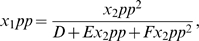
(5)where the constants 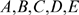 and 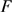 are combinations of the rate and binding constants; they are not presented here for brevity. Since these terms describe the activation of MAPKKK by input signal (equation 3), MAPKK by MAPKKK (equation 4), and of MAPK by MAPKK (equation 5), to determine the overall order of activation of MAPK by input signal we substitute equations 3 and 4 into equation 5, and can see that the overall system is described by a function similar in nature to a 4th order Hill equation.

Secondly, if we assume that 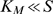, then the MM term simplifies as follows:
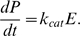
This limiting case corresponds to very tight enzyme-substrate interactions. By applying the tight enzyme-substrate assumption to the phosphorylation reactions, and again the weak enzyme substrate assumption to the dephosphorylation, we find:
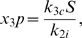
(6)

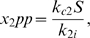
(7)and
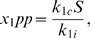
(8)which corresponds to linear increase in the amount of output signal as a function of the input. However, in reality, this scenario will never hold for all values of 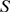; rather, the amount of output signal will increase as a linear function of 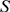 until 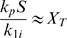, the total amount of enzyme the system, at which point no further increase is possible and the signal output plateaus. In this situation, the signal output graph will resemble a Hill function with an exponent of approximately 1.

The first assumption (weak interactions) can be easy to justify over the course of a reaction: if no new substrate is created, and if the binding constant has a much higher value than the initial substrate levels, then the binding constant will always be much greater than the substrate levels. On the other hand, the second assumption (tight interactions) is more drastic and can likely be violated during the reaction duration. Nonetheless, these two simplifications provide useful analytic bounds with which to describe the nature of the system output. We term the parameter regime which favors outputs of the nature of equation 5 to be the ‘weak’ regime, and that which favors equation 8 to be the ‘tight’ regime.

#### Numerical analysis of Hill coefficient distributions with and without scaffolds

To study signal-response sensitivity further, we simulated model (1) for many different randomly selected values of certain parameters. We pick coefficients 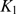 and 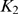 as 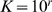, where 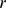 is a uniformly distributed random number in [−3,3]. All other parameters are taken as in table 2. We record the steady state level of 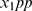 as a function of signal input strength 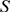 and use a nonlinear least squares regression algorithm to fit the data to the Hill equation described above (in accordance with past theoretical investigations). We repeat this process 10000 times, to measure the spread of possible Hill coefficients the model is capable of generating. It is important to note here that the literature value for the binding constant of the phosphatase enzymes, which catalyze the backwards reactions, are much smaller than necessary to impose the weak binding approximation. Thus, we expect in our simulations for there to be higher rates of inactivation reactions, and therefore a potential for higher Hill coefficients than predicted analytically.

**Figure 4:**
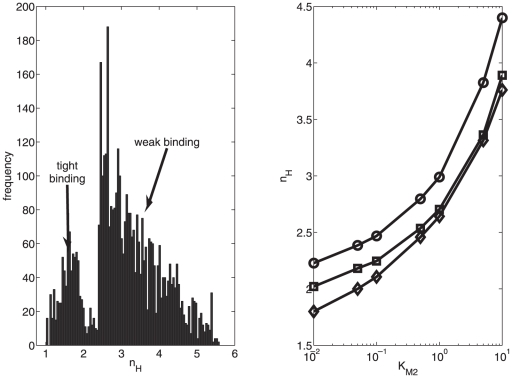
Dependence of the Hill coefficient on Michaelis-Menton binding constants in the canonical MAPK pathway. (left) Spread of Hill coefficients calculated from model (1). Exponents for binding and kinetic constants for MAPK and MAPKK were drawn from a uniform (−3,3) distribution. (right) Representative Hill plots for model (1). Kinetic constants are 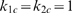. Lines represent 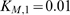 (diamonds), 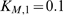 (squares), and 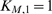 (circles).

As seen in the left panel of figure 4, there are two regimes of possible Hill coefficients, which correspond to parameters from the tight and weak regimes, as labeled. It is interesting to note that these regimes are quite distinct in terms of their output; there is a significant gap between the weak (Hill coefficient greater than 2.5) and the tight (Hill coefficient less than 2.0) parameter regimes, with very little ‘mixing’ evident.

We next take a small sample of these parameter sets and determine the relationship between 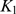, 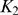 and the Hill coefficient. As plotted in the right panel of 4, the net ultrasensitivity of the pathway output relative to input signal increases as both binding parameters increase in value. This coincides with our analytical predictions for the two limiting cases of very small and very large values of the binding parameters. We conclude that this pathway can be configured to exhibit either a graded response (for sufficiently low binding constants) or an ultrasensitive one.

This result is very interesting in light of what is known about the yeast pheromone pathway. The MAPKK-MAPK interaction is reported to be quite tight [1], with binding constants of less than 100 nM. From figure 4, this would imply a graded response to activation of MAPKK. However, the MAPKKK-MAPKK interaction is very weak, requiring a scaffold for stabilization. So, we next turn to the role the scaffold plays in shaping the output signal.

**Figure 5:**
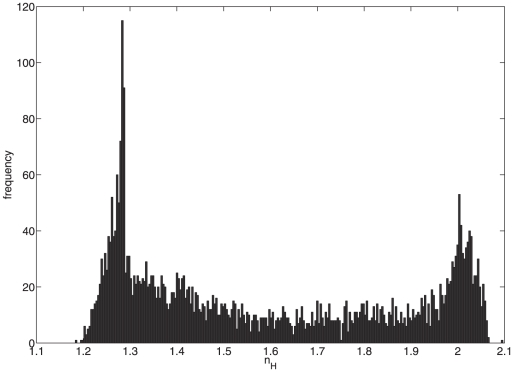
Distribution of Hill coefficients of model 2 given binding constants (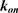, 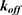) with uniform log distributions.

We perform a similar numerical experiment, over a wide range of possible binding constant values, on the with-scaffold system (model 2). We randomly calculate these constants by drawing them from a uniform log distribution (i.e., we draw their base 10 exponent from a uniform distribution) spanning from 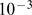 to 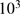. We then simulate the model for increasing signal input strengths to generate a Hill profile and use nonlinear least squares regression analysis to compute the Hill coefficient. The distribution of Hill coefficients thus calculated is shown in figure 5.

As seen in figure 5, the model is capable of reproducing Hill profiles with a range of Hill coefficients spanning the biologically relevant range of 1.5–2.1 (based on the available yeast data), indicating that the model, with proper parameterization, is capable of describing the biological system.

### Scaffold and response sensitivity: modeling experimental systems

The numerical surveys of both the with-scaffold (figure 4) and without-scaffold (figure 5) models demonstrate that these systems have the capacity to properly reflect the experimental behavior of the yeast pheromone signaling system. The recent papers by Hao *et al* [12] and Takahashi and Pryciak [19] both incorporate experiments in which the signal-scaffold-MAPK unit is disrupted. In the former, a mutant scaffold deficient in Fus3 MAPK binding is introduced. Upon addition of pheromone, this system displays a more graded profile for MAPK output. In the latter, a mutant, constitutively active Ste20 MAPKKK is introduced, leading to a permanent signal independent of pheromone induction. The Fus3 MAPK output signal is significantly more ultrasensitive to increases in this mutant Ste20 MAPKKK expression level in the presence of the scaffold than in its absence. In this section, we attempt to explain all of these findings by using models (1) and (2). The key is how each of these experiments changes the relative binding rates for the proteins in the complexes.

#### Signal independent Ste5 alignment abrogates ultrasensitivity

An important distinction between the mammalian and yeast scaffold-MAPK systems is the requirement, in yeast, for membrane recruitment of the scaffold prior to full scaffold activation. Mammalian scaffolds, such as the MP1 protein, do not have such a requirement for proper activation [8], [20]. The mammalian system, as analyzed numerically by Levchenko *et al*, demonstrates a significantly different response profile than the yeast system [8]. Specifically, it was shown that the mammalian scaffolds lead to a strongly graded signal response under biologically relevant parameterization; only through extremely restrictive parameter choices could an ultrasensitive response be measured, and that response had an overall output strength many orders of magnitude lower than the more favored graded response.

**Figure 6:**
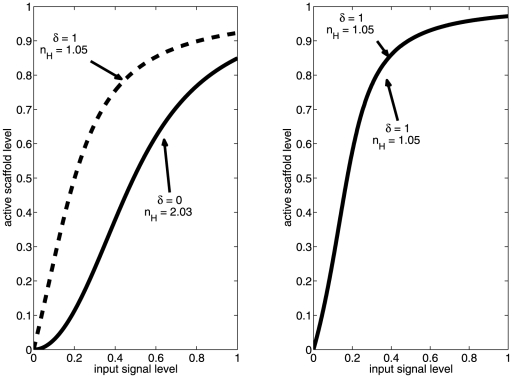
Signal response of the scaffold-MAPK complex in the presence (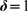) or absence (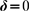) of constitutive 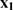 binding. Plots represent slow (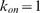, left panel) or fast (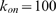, right panel) scaffold-membrane association rates.

With the results from the mammalian system in mind, we next explore what role the selective activation hypothesis plays in shaping the response curve. We modify the model by allowing scaffold realignment, the 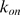 reaction in model 2, to occur in the absence of signal with a flag parameter 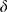. Here 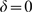 corresponds to the yeast wild type configuration, and 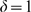 allows for signal-competent scaffold to always exist in the cell, which is more representative of mammalian scaffolds. As seen in figure 6, loss of the selective activation component of scaffold activation results in the loss of ultrasensitive behavior in the response, in accordance with the theoretical investigations of Levchenko.

This result is a strong theoretical indicator for the selective activation hypothesis. The one extra step involved in selective activation, the recruitment of the scaffold to the plasma membrane prior to formation of signal-competent scaffold, is critically involved in the formation of the ultrasensitive response signal. In light of this result, we can now begin to analyze and understand the both studies of Takahashi and Pryciak, in which signal is artificially induced at the Ste11 MAPKKK level while bypassing the receptor; and the studies of Hao *et al*, which showed that loss of Fus3-Ste5 association converted the Fus3 response to a more Kss1-like response.

#### The role of scaffold localization in signal response

The paper by Takahashi and Pryciak employs a Fus3-driven reporter gene to measure pathway activation [19]. In this set of experiments, inducible genes are used to express mutant variants of several elements of the MAPK cascade. Here we focus on one type of such mutants. The authors express a form of the Ste5 scaffold which is permanently attached to the plasma membrane. They find that the tethered scaffold leads to a graded response in the downstream gene expression reporter [19].

**Figure 7:**
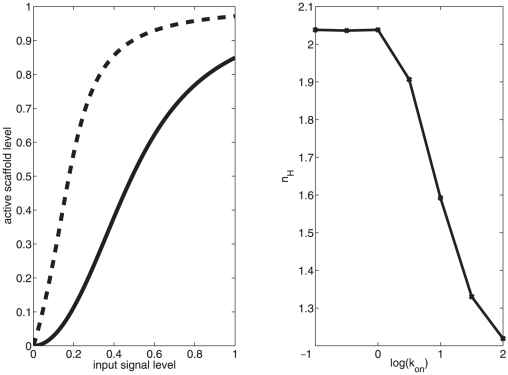
Signal response of the scaffold-MAPK complex as a function of scaffold-membrane binding and alignment rate. (left) Representative Hill plots for 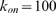 (dashed) and 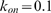 (solid). (right) Dependence of the Hill coefficient on 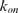. All other parameters as in table 4.

We can use model (2) to model this experimental system and gain understanding of how membrane binding affects ultrasensitivity. By varying the complex-membrane association rate 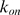 we can simulate a scaffold which is either tethered to the membrane (high 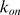) or a scaffold which is free to dissociate and diffuse throughout the cytoplasm (low 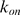). As seen in figure 7, increasing 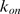 leads to a decrease in the Hill coefficient and therefore a more graded signal response.

From these simulations, we observe that a scaffold with enhanced membrane binding ability signals with a graded response to input stimulus, whereas a diffusible scaffold signals with an ultrasensitive response. This mirrors the result from Takahashi and Pryciak, if we assume that the natural effect of tethering all the scaffold complexes to the plasma membrane is to increase scaffold-signal association rate, by forcing the two components into much closer long-term proximity.

**Figure 8:**
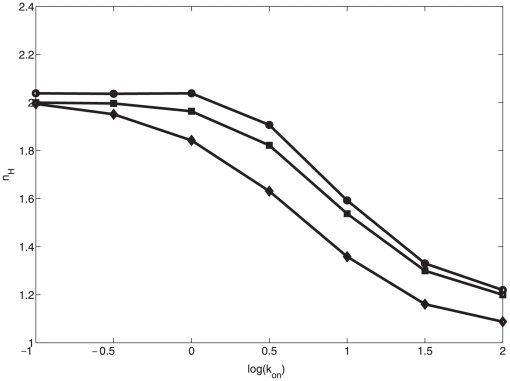
Dependence of ultrasensitive output response on cytosolic phosphorylation of scaffolds. Plots of Hill coefficient as a function of 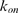 are shown for cytosolic rate control parameter 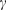 = 0 (circles), 0.1 (squares) and 1 (diamonds).

We have already observed that loss of the requirement for signal-induced membrane binding prior to full scaffold activation leads to a decrease in the ultrasensitive nature of the signal response. We now look to determine whether it is the scaffold alignment or initial enzymatic activation of Ste11 which is responsible for the majority of the ultrasensitive behavior. We perform a numerical experiment in which any scaffold, whether bound to the membrane or free in the cytoplasm, can undergo the enzymatic activation step. The relative level of cytosolic scaffold phosphorylation is controlled by the parameter 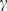; 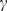 = 0 implies the scaffold must be bound to the membrane for activation, while 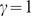 means that cytosolic scaffold is targeted at the same rate as membrane bound scaffold. The results of this experiment are presented in figure 8. In this experiment, we observe a very minor decrease in ultrasensitive response from the scaffold even in the case where the cytosolic scaffold is as strong a phosphorylation target as is the membrane bound scaffold.

Thus, in this system it is clear that the alignment of Ste11, Ste7 and Fus3 on the scaffold is the critical step needed to promote an ultrasensitive response.

#### Selective activation of Ste5 controls ultrasensitivity

Next, we consider a second experiment by Takahashi and Pryciak [19]. The authors express a form of the Ste11 MAPKKK enzyme which is always active. Thus, the scaffold never needs to associate with the plasma membrane to become active. In the case of the constitutively active Ste11, the resulting output signal is ultrasensitive. Moreover, presence of the Ste5 scaffold increases this ultrasensitivity by a factor of nearly 2 [19].

We again note here that the results of this experiment contradict current theoretical understanding of how the MAPK pathway functions in the presence and absence of scaffolds. A pathway with a scaffold based on the mammalian MAPK system, as modeled by Levchenko *et al*, showed a strong tendency for a graded response [8], as did our model with the removal of selective activation (fig 6). On the other hand, according to the results of Takahashi and Pryciak, expression of active Ste11 in the absence of Ste5 scaffold resulted in a lower degree of ultrasensitivity than in the presence of Ste5. Here we provide a hypothesis which might explain the mechanism underlying this observation.

In this system, there is no requirement for extracellular pheromone, so we assume the scaffold remains in its locked, non-permissive configuration (fig 3, no 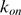 reactions). However, since Ste11 is active, this complex itself has enzymatic activity for Ste7 MAPKK. Thus, despite the fact that the enzymes are all associated with the scaffold, the system is more properly described by model (1), in which the Ste5 complex is both the enzyme (because Ste11 is active) and the substrate (because Ste7 is attached but misaligned). As reported in the literature [1], [2], the Ste11-Ste7 interaction is weak, with a very high Michaelis-Menton constant. As seen in figure 4 and equation (5), this would produce a very strongly ultrasensitive response. On the other hand, the Ste7-Fus3 interaction is quite strong [1], causing a lower Hill coefficient and therefore a more graded response (figure 4 and equation (8)). Taken together, we would expect the full pathway to exhibit mild to moderate ultrasensitivity (Hill coefficient above 2 but less than 4). And, as seen in [19], the MAPK system with dominantly active ste11 and without scaffold shows a Hill coefficient of 2. The question now remains how to explain the role the scaffold plays in this particular situation of dramatically increasing the Hill coefficient to a value of 4.

Our results suggest the following hypothesis. Scaffolds in the cytoplasm will assemble into their resting state, a noncompetent form; however, now they have dominantly active ste11 attached instead of wild-type, signal-dependent ste11. There are two possible modes of full activation: either the scaffolds transmit the signal in *cis* (from a scaffold's ste11 to its ste7 to its fus3), or in *trans* (from ste11 of one scaffold to ste7 of another, and from that ste7 to the fus3 of another scaffold) by one scaffold acting as an enzyme transmitting the signal to another scaffold, which serves as the substrate.

Using model 2 as a basis, we can show that *cis* transmission is not responsible for the marked increase in Hill coefficient. Consider the scenario in which a misaligned scaffold can still transmit signal from Ste7 to Fus3 at some basal leakage rate. Then, based on the combinatorial nomenclature previously introduced, we can remove two of the three binary naming states (Ste11 is never dephosphorylated and the scaffold is never aligned) reducing the system to two dynamic variables, 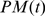 and 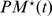 connected by only two reactions, the leakage activation reaction occurring at rate 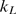 and the standard dephosphorylation inactivation reaction. If we further assume that the total amount of scaffold-associated dominantly active Ste11 is a function only of its induction signal (that is, 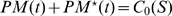 for some appropriate function 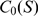), the two variable system can be described by a single ODE:
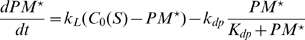
(9)This equation has an equilibrium value of:
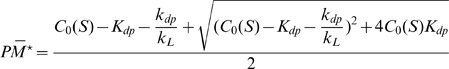
If we define 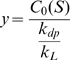, substitute into the steady state expression and expand for small values of 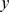, we can make the critical observation that this steady state is linear with respect to the total amount of dominantly active Ste11 in the system. Note this expansion is justified since we have already assumed that the leakage rate, 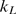, is very small. The only mechanism by which a Hill coefficient of 4 could be observed is if the induction mechanism for the gene were to obey a fourth order Hill law; since the same induction mechanism yields an overall Hill coefficient of 2 in the no scaffold case, we can reject that possibility.

**Figure 9:**
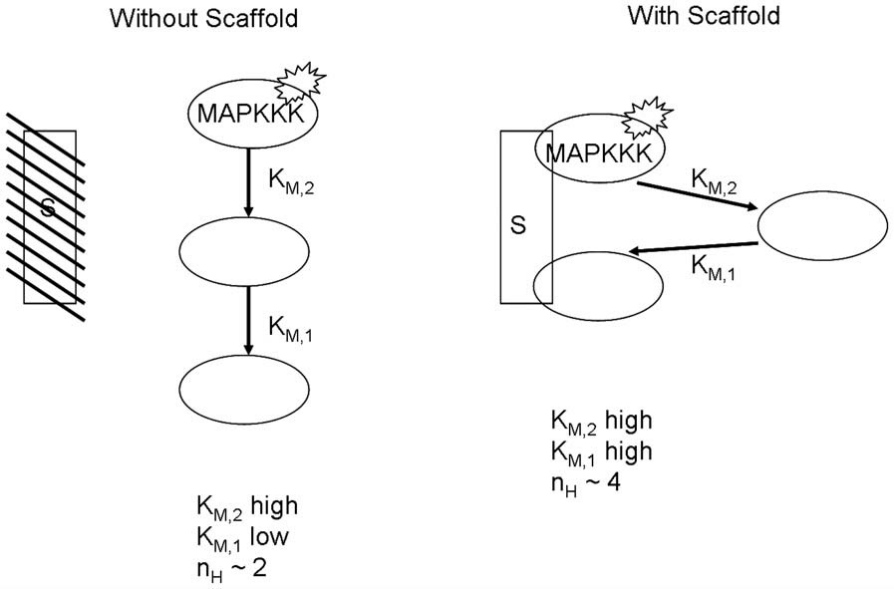
Schematic representation of signal transduction with constitutive MAPKKK activity in the absence (left) and presence (right) of scaffold.

Therefore, under our hypothesis concerning selective activation, the scaffolds in the no signal, dominantly active Ste11 case must transmit their information in *trans*. The difference in Hill coefficient is then a consequence of difference in binding constants of the scaffold-associated versus scaffold-free MAPK enzymes. We would predict that the steric constraints of the fully assembled–but misaligned–scaffold would exhibit very weak enzymatic associations between the components of the pathway, leading to very large binding constants and therefore a very high Hill coefficient (see figure 4). Conversely, in the absence of scaffold, the system behaves as put forth in model 1, which we have shown can exhibit total Hill coefficients in the range of 2 (figure 4). We summarize this hypothesis schematically in figure 9.

To test this hypothesis, we envision the following experiment. Consider the induction of the dominantly activated ste11 in a cell expressing a variant of ste5 incapable of binding one of its three targets. If our assumption about *trans*-acting scaffolds suffering steric effects is accurate, ablating any one of the three enzymatic binding sites should significantly reduce these steric effects, allowing for the enzymes to more closely approach their scaffold-free association rates and thus lead to a decrease in the Hill coefficient.

To test the hypothesis that it is not just membrane localization, but selective activation at the membrane, which leads to ultrasensitivity, we propose this experiment. Consider the situation of co-expression of the tethered Ste5 scaffold with the dominantly active Ste11. If our hypothesis is correct, this system will still demonstrate a strong ultrasensitive character, as there is no external signal through the receptor to permit selective activation. We cannot predict the exact value of the Hill coefficient, as there will be new competing forces acting on the system (localization to the membrane will create an artificially increased concentration of scaffolds, but tethering may increase the steric difficulties of *trans* signalling). On the other hand, if pure localization is the key determinant in ultrasensitivity, then this joint system will revert to a graded signal response.

#### Selective activation rate modulates the output signal character

In the paper by Hao *et al*, the authors employ a microfluidic device to create specific gradients of the yeast 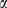 factor, and observe how the behavior of the yeast cell changes as a function of factor concentration [12]. They also harvest cells from each distinct zone of behavior (vegetative growth, elongated growth, pre-mating growth) and determine the level of activation of each of the 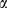 factor responsive kinases, Fus3 and Kss1. They find that Kss1 exhibits a graded response to increases in factor concentration, whereas Fus3 shows an ultrasensitive response. Further, upon expressing a mutant allele of the Ste5 scaffold which has a much lower binding affinity for Fus3, the ultrasensitive response of Fus3 is converted to a Kss1-like graded response.

Again, the experimental results contradict the existing theory: the canonical result from Huang and Ferrell is that an unscaffolded pathway response should be ultrasensitive [13], and the MAPK system with a scaffold tends to show a graded response [8]. Yet the experimental results from the yeast system show the opposite effect: the Fus3 protein, which depends critically on the presence of its scaffold, has an ultrasensitive response, whereas the Kss1 protein, which functions without the scaffold, has a graded response.

**Figure 10:**
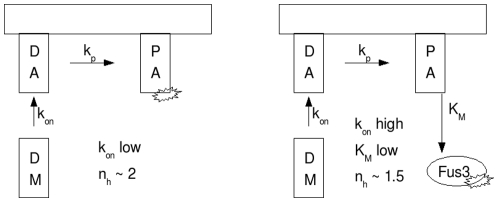
Schematic representation of yeast pheromone signal transduction with proper (left) and abrogated (right) Fus3-Ste5 interaction.

We now consider the mutant scaffold, which is able to bind Ste11 and Ste7, but not Fus3. In their report detailing the role Ste5 plays in modulating the Fus3 response to pheromone, Hao et al present a simple model describing how adjustment of the catalytic rate and binding constants may allow a cascade with two possible end targets (in the case of yeast, Fus3 and Kss1) to generate both an ultrasensitive (Fus3) and graded (Kss1) response. However, in their model, the scaffold itself is not addressed, which we can now rectify. This system is still dependent upon addition of pheromone, and so we assume the enzyme-scaffold binding reactions occur as described in model (2) previously. Once pheromone is added, the final complex formed involves active Ste11 and active Ste7. The issue is how this system is now less ultrasensitive than the wild type, as it involves all the key steps identified earlier as components of the ultrasensitive nature of the signal. The results in figure 4 and 7 suggest a hypothesis which resolves the issue. As previously mentioned, Ste7 on an active scaffold has very high enzymatic affinity for its MAPK targets Fus3 and Kss1 [14], and so we expect a more graded response of MAPK activation in response to Ste7 activation (figure 4 and equation (8)). Moreover, the scaffold demonstrated a significant decrease in ultrasensitive character as the activation rate, 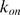, was increased (figure 7). Taken together, we hypothesize that the MAPK-binding deficient scaffold has a higher Ste7 assembly rate than the wild type scaffold, perhaps due to the Ste7 binding site being easier to reach without Fus3 present. This hypothesis is presented diagrammically in figure 10.

**Figure 11:**
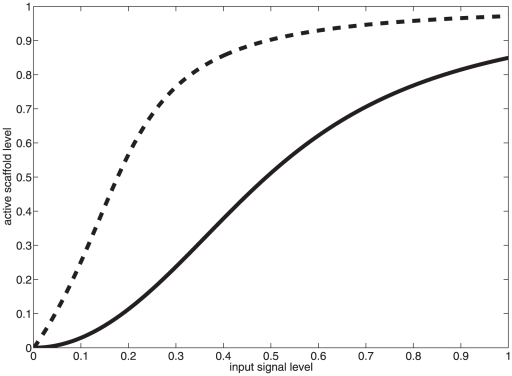
Abrogation of Fus3-scaffold interaction can lead to loss of ultrasensitive Fus3 response. Wild-type (solid, 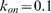, total activated scaffold) and Fus3-less (dashed, 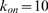, free active Fus3) scaffold system responses are plotted for 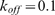.

To test the plausibility of this hypothesis, we modify our model to ablate the fus3 binding site. We assume instead that fus3 is present in the cytoplasm and interacts via standard Michaelis-Menton kinetics with activated, scaffold-associated ste7. We then simulate the model under two conditions: first, without modification with a slow 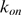 rate; second, with fus3 activation occurring off-scaffold, but with the scaffold having a much faster selective activation rate. For our hypothesis to be plausible, we must be able to observe a lower Hill coefficient in the second system than in the first. The results of this simulation are plotted in figure 11. We clearly observe a proof-of-concept, in that a system in which the scaffold is unable to bind Fus3 but can align ste11 and ste7 much more quickly, is in fact capable of generating a more graded response. Beyond proof of concept, it is also important to note that our hypothesis here predicts that Fus3 should reach its response maximum faster in the scenario in which it does not bind to the scaffold, due to a significantly decreased time to ste7 activation. It has been observed that in wild type systems, scaffold-associated Fus3 is considerably slower to reach its activity maximum than scaffold-free Kss1, and in the experiment in which Fus3 cannot bind to the scaffold, its activation kinetics mirror much more closely the faster Kss1 rates [12].

## Discussion

In this work, we have formulated two complimentary models of the yeast pheromone response pathway, in the absence (model (1)) and the presence (model (2)) of the Ste5 scaffold. The first model is a revisit of the original MAPK model first discussed by Ferrel, while the second is a simplified system describing the influence of the Ste5 scaffold on the MAPK pathway. We show how in both cases a careful examination of the binding constants, which dictate how strongly the enzymes associate with one another, lead to results consistent with recent experimented observations. In particular, we describe how manipulation of the protein-protein binding constants can lead to a multitude of signal response profiles, trending from strongly graded to sharply ultrasensitive.

Specifically we highlight the following:

1. **Selective scaffold activation modulates signal output.** Based on the results of our models, the dual requirements of Ste5 binding to the plasma membrane and phosphorylative activation of Ste11 to jointly induce MAPK signaling is crucial for allowing the system to exhibit both graded and ultrasensitive response profiles. The extent to which the selective activation step is able to shift the signal response from the scaffold ‘default’ of graded response to a switch-like ultrasensitive profile is critically dependent upon the protein-protein interaction strengths of the components in the system. In particular, stronger association of the scaffold with its activator decreases the ultrasensitivity of the system, as does stronger association of the selectively activated component with the scaffold.
2. **Prediction of global system changes based on local changes.** The experimental papers under consideration in the formulation of these models both involved biochemical manipulations of the pheromone response pathway, leading to changes in the signal response. We have shown, by coupling the results of our simulations with the results of these manipulations, how mathematical models can be used to predict wide-ranging effects caused by these small perturbations of the original system. We have presented several hypotheses concerning how a particular change in the system–such as constitutive Ste11 activity or abrogation of Fus3-Ste5 interaction–leads to global changes in the system so that the observed signal response might be formed.

### Module Analysis of Signaling Networks

A common criticism of mathematical analysis of biological networks is that, for standard analytic techniques to be applied, the system must be simplified to such a great extent that is must lose various important, complex details, rendering the results of the analysis suspect at best. However, we have shown how even a fairly simple model, model (2), is capable of suggesting general hypotheses about the nature of systemwide interactions based on a single perturbation event. This lends credence to the idea of intelligent reductionism, a feature of systems biology. Rather than reduce a complex system into all its possible individual components and study each in isolation, a complex system can instead be broken into reasonably independent modules, and each module studied alone and with regulatory interactions with other modules. This approach has been successfully applied to the study of receptor tyrosine kinases [21], the mammalian MAPK-immediate early gene systems [22], and other signaling networks [23], [24]. It is also suggestive that analysis of such network modules can be useful in understanding exactly how experimental manipulation of a system at a single focus point leads to other global changes in network response pathways.
